# Dynamic Analyses of Transcriptome and Metabolic Profiling: Revealing Molecular Insight of Aroma Synthesis of Mango (*Mangifera indica* L. Var. Tainong)

**DOI:** 10.3389/fpls.2021.666805

**Published:** 2021-05-07

**Authors:** Ming Xin, Changbao Li, Hock Eng Khoo, Li Li, Xuemei He, Ping Yi, Yayuan Tang, Jian Sun

**Affiliations:** ^1^Agro-food Science and Technology Research Institute, Guangxi Academy of Agricultural Sciences, Nanning, China; ^2^College of Chemistry and Bioengineering, Guilin University of Technology, Guilin, China; ^3^Guangxi Key Laboratory of Fruits and Vegetables Storage-processing Technology, Guangxi Academy of Agricultural Sciences, Nanning, China

**Keywords:** aromatic compound, de novo transcriptome assembly, gene ontology, RT-PCR, volatile profile

## Abstract

This study aimed to evaluate the changes in aromatic components and other chemical properties of Tainong mango during fruit development, ripening, and storage. As the volatiles of Tainong mango and their related molecular mechanisms remain unclear, volatile profile, metabonomics, and transcriptome analyses were applied to investigate the molecular determinants of the synthesis of aroma components in mango during fruit development and storage. Total acids, total sugar, total carotenoids, enzyme activities of the mango pulp samples were also determined. Volatile components of the mango pulp samples were identified using a gas chromatography-mass spectrometric method. Ribonucleic acid (RNA) sequences of the samples were analyzed by real-time polymerase chain reaction. The results showed that 181 volatiles were isolated and identified in the fruit at seven stages. Compared to the other stages, mango collected on day 8 and day 12 had higher concentrations of 17 volatile components, especially (*E*,*Z*)-2,6-nonadienal, 53384 transcripts were also detected through RNA sequencing. The differentially expressed genes analyses included catalytic activity, transferase activity, adenosine diphosphate binding, transcription factor activity, and oxidoreductase activity. α-Pinene content and expression of the differentially expressed genes involved in terpenoid metabolism and enzyme activities in the terpenoid metabolic pathways gradually increased during the maturity of the fruit, and had maximum values at day 8 of storage. Moreover, the integrative analyses revealed potential molecular insights of mango development and aroma formation in the fruit.

## Introduction

Mango (*Mangifera indica* L.), the king of tropical fruit, is native to South Asia (Munafo et al., 2014). It is particularly rich in β-carotene. The compound is a precursor of vitamin A, which is rare in many fruits. Mango pulp is a typical source for the productions of fruit jam, canned food, pickled, sour, spicy pickles, and beverages, and more, besides being suitable for immediate consumption. It also exhibits tempting fragrance derived from the volatile components (San et al., 2017). The quality and acceptability of mango pulp and juice are mainly assessed based on the flavor (Zhang et al., 2019a). The volatile profile varies considerably among different mango cultivars.

To date, a broad scope of investigations on mango volatiles has been implemented, resulting in the isolating and identification of more than 400 compounds in different varieties of mango, most of which are esters, ketones, aldehydes, alcohols, and terpenes (Andrade et al., 2000; Pino and Mesa, 2006; Shivashankara et al., 2006). Literature has shown that a relatively high amount of α-terpinolene has been determined in Kensington Pride, Chana, Bacuri, Coquinho, Gojoba, Cametá, Cheiro, Comum, and Carlota mangoes (Andrade et al., 2000; Lalel et al., 2003). It is one of the main volatiles in these fruits. A previous study also reported that 4-methoxy-2,5-dimethyl-3(2H)-furanone (MDMF), β-damascenone, ethyl butanoate, ethyl hexanoate, (*R*)-linalool, (*E*,*Z*)-2,6-nonadienal, and terpinolene are the essential aromatic compounds determined in yellow Thai Keaw mangoes (Boonbumrung et al., 2001). Hence, ethyl butanoate is one of the important aromatic compounds found in different cultivars of mango grown in Brazil (Tommy Atkins, Coracao de Boi, Carlota, Rubi, Haden, and Espada). Moreover, (*E*)-2-decenal, hexanal, (*E*)-β-ocimene, (*E*)-2-hexenal, γ-terpinene and (*Z*)-3-hexenal have been detected as the main aromatic components in Thai Khieo Sawoei mangoes (Tamura et al., 2001).

Based on the odor activity values, which represents the ratio of concentration to odor threshold, MDMF, ethyl butanoate, methyl benzoate, ethyl 2-methylpropanoate, decanal, (*E*,*Z*)-2,6-nonadienal, (*E*)-β-ionone, and (*E*)-2-nonenal have been potentially identified as the most important aroma compounds in the 20 cultivars of mango (Pino and Mesa, 2006). Literature also shows that terpinolene, (*E*,*Z*)-2,6-nonadienal, (*E*)-β-ionone, β-damascenone, (*E*)-2-nonenal, ethyl butanoate, and ethyl 2-methylpropanoate are the odor-active compounds of Corazon mangoes (Pino, 2012). In addition to the small number of these aromatic compounds identified in the mango pulp samples, many other volatiles could not be quantified due to their low detection limits (Zhang et al., 2019a).

The maturity of mango had greatly affected the aroma profile of the fruit besides the geographical origin, which was related to the growing environment and the variation in cultivar (Lebrun et al., 2008; Pandit et al., 2009b; Chauhan et al., 2010; Kulkarni et al., 2012). A previous study identified limonene and *p*-cymene, α-terpinene, and ethyl octanoate as the dominant volatile components in Kensington Pride mango samples collected during the pre-climacteric, climacteric stage, and fully ripe stages (Lalel et al., 2003). Although many volatile components have been isolated and identified in mangoes, studies on the molecular mechanism of aroma compound biosynthesis are limited. Acyl-CoA-oxidase (MiACO), 9-lipoxygenase (Mi9LOX), hydroperoxide lyase (MiHPL), peroxygenase (MiPGX1), and epoxide hydrolase 2 (MiEH2) genes involved in lactone biosynthesis have also been isolated from mango, and the transcript profiling of these genes was analyzed during various developmental stages in fruits of three mango cultivars (Kent, Pairi, and Alphonso) with different levels of lactones (Deshpande et al., 2017b). The transcriptome analysis has also been used to explore the distinct aroma characteristics in Alphonso mango, where transcripts for the biosynthesis of furanones, sesqui-terpenes, lactones, monoterpenes, and diterpenes were identified.

Aroma is the most critical organoleptic quality of a mango. Volatile components give aroma to the fruit. These compounds are subjected to changes during fruit ripening and postharvesting. Although volatile components of fruits are widely studied, the associated molecular mechanisms remain unclear. It undergoes rapid and substantial changes during ripening and storage. Although some of the aromatic components have been isolated and identified in different cultivars of mango, little is known about the volatile profile in the “Tainong” variety of mango, which is widely grown in southern China. The ripe fruit of this variety has a special aroma because it contains certain aromatic compounds that give it a flavor. In this study, an integrative analysis of volatile profile and transcriptome was employed to identify molecular mechanism, as well as the changes in volatile components during the stages of fruit development and storage.

## Materials and Methods

### Plant Materials and Growth Conditions

Tainong mango trees have been planted in the Tiandong National Mango Germplasm Resources Nursery in Guangxi (Tiandong County, Baise City, Guangxi Province, China, 23°16′N, 107°26′E) since 6 years ago. The ambient temperatures of the nursery ranged between 25 and 28°C, and the altitude is 110 m. Mango samples were collected from the nursery at different stages of fruit development (40, 60, 80, and 90 days after the flowering stage began). Fruit samples harvested at 90 days after the flowering were also kept for 4, 8, and 12 days in the laboratory at a controlled temperature of 25°C and a humidity level of 95%. Seven mangoes of an average weight of 100 ± 10 g were sampled. The fruits were picked from different trees at the same positions on the day of fruit collection. Mango peels were removed, and the pulps were directly used for volatile analysis. All pulp samples were stored at –80°C before extraction. Data of triplicate analyses were obtained for each analysis.

### Analysis of Total Acid Content, Total Sugar Content, and Carotenoid Content

Total acid content, total sugar content, and carotenoid content of the mango pulp samples were measured according to the methods described by Shi et al. (2013); Liao et al. (2019), and Zhang et al. (2019a), respectively. The total acid content of mango pulp samples was determined by following the Official Methods of Analysis for Vinegar in China (GB/T 5009.41-2003), while total sugars and total carotenoids of the pulp were determined using HPLC.

### Determination of Enzyme Activities

Enzyme activities of 1-deoxy-D-xylose-5-phosphate reductase (DXS), 1-deoxyxylose-5-phosphate synthase (DXR), geranyl pyrophosphate synthetase (GPPS), geranylgeranyl pyrophosphate synthetase (GGPPS), pyruvate carboxylase (PC), diacylglyceryl transferase (DGAT), farnesyl diphosphate synthase (FPS), and hydroxymethyl glutarate monoacyl CoA reductase (HGMR) were measured using plant ELISA kits, where the all the ELISA kits were purchased from a local chemical supplier (Jianglai Biotechnology Co., Ltd, Shanghai, China). A microplate reader (BioTek Instruments, Inc., Winooski, VT, United States) was used to obtain the absorbance readings of each test; the analyses were performed according to the instructions provided by the manufacturers. The results were calculated based on the formula provided in the manufacturers’ instructions.

### Volatile Profile Analysis

Exactly 2.5 g of homogenized mango pulp was added with 2.5 mL of saturated sodium chloride and 100 μL of internal standard solution (32.88 μg/mL 2-octanol, Sigma-Aldrich) in a 20 mL headspace bottle (ANPEL Laboratory Technologies Inc., Shanghai, China). The bottle was sealed with a crew cap fitted with a PTFE/silicone septum. After a 15-min agitation at 50°C and 250 rpm, volatile compounds were extracted with 50/30 μm DVB/CAR/PDMS as the extraction fiber, which was later exposed to the headspace for 40 min.

The gas chromatography-mass spectrometry (GC-MS) analysis was performed using a GC-mass spectrometer (7890B-5977B, Agilent Technologies) to quantify the volatile components of mango pulp samples. Separation of the volatile compounds was performed using a DB-Wax column (30 m × 0.25 mm × 0.25 μm, Agilent Technologies, Shanghai, China). The extract was injected in splitless mode and desorbed at 260°C for 5 min. Helium was used as the carrier gas with a constant flow rate of 1 mL/min. The initial oven temperature was 40°C, and then programmed at 5°C/min to 220°C, followed by an increase of 20°C/min to 250°C, and finally held at 250°C for another 2.5 min. Electron ionization (EI+) was set at 70 eV, and the data were recorded in scan mode of *m*/*z* 20–400.

Based on the MS fragmentation patterns and linear retention indices, volatile compounds were identified and quantified through comparisons with the NIST14 library. The differential volatiles in each group were screened according to the following criteria: fold change ≥ 1.5 or fold change ≤ 0.67; variable importance in project (VIP) ≥ 1.

### Real-Time PCR Analysis

The real-time quantitative PCR (qRT-PCR) was performed using the fluorescent intercalating dye SYBR Green in a detection system (MJ Research, Opticon 2), and MiACT was used as a standard control (Luo et al., 2013). The two-step RT-PCR procedure was performed according to the method described by Li et al. (2005).

### Ribonucleic Acid Sequencing

RNAprep pure plant plus kit (TIANGEN Biotech Co., Ltd., Beijing China) was used to purify total ribonucleic acid (RNA) in mango pulp samples. The purification steps were done in accordance with the manufacturer’s instructions. After a quality checked using NanoPhotometer^®^ spectrophotometer and Agilent 2100 bioanalyzer, high-quality mRNA was enriched by poly-T oligo-attached magnetic beads. The library was constructed using the NEBNext^®^ Ultra^TM^ RNA Library Prep Kit for Illumina^®^ (NEB, United States). After the fragmentation of the purified mRNA by divalent cations at elevated temperature, the first-strand complementary DNA (cDNA) was generated using a random hexamer primer. The second strand of cDNA was generated using DNA polymerase I of M-MuLV reverse transcriptase (RNase H-) and RNase H (Sigma-Aldrich, Shanghai, China). After methylating the 3’ ends of DNA fragments and ligating the adaptor for hybridization, the library fragments were purified using AMPure XP beads (Beckman Coulter, Beverly, United States). Under the action of high-fidelity DNA polymerase, Universal PCR primers, and Index (X) primer, PCR was performed, and the PCR products were purified with AMPure XP system. The list of primers used is showed in Supplementary Table 1. Then, match quality of the library was assessed using the Agilent 2100 bioanalyzer system (Waldbronn, Germany). The library sequencing was performed using the Illumina HiSeq 2500^TM^ platform (PE125, paired-end).

### *De novo* Transcriptome Assembly, Gene Expression, and Differential Expression Analysis

After the quality check and adaptor trimming, clean reads were assembled using the Trinity software, and the transcripts were generated. The reads contained unknown and over 50% low-quality nucleotides (Qphred ≤ 20) were removed. Quality of the transcripts was evaluated using the Benchmarking Universal Single-Copy Orthologs. The coding sequences (CDs) were predicted through comparisons with the NR and Swissprot protein libraries, and using ESTScan v3.0.3 software.

The clean reads were mapped to the transcripts using RSEM v1.1.17 software (Li and Dewey, 2011). The gene expression level was quantified using FPKM (fragments per kilobase of transcript per million fragments mapped) as an indicator. FPKM was calculated as follows:

(1) $$\text{FPKM}=\frac{\text{Mapped}\,\text{fragments}\,\text{of}\,\text{transcript}}{\text{Total}\,\text{count}\,\text{of}\,\text{mapped}\,\text{fragments}\,\left(\text{millions}\right)\times \text{Length}\,\text{of}\,\text{transcript}\,\left(\text{kb}\right)}$$

Differential expression analysis was done using the DEGSeq2, which was according to the criteria as follows: log_2_ (fold change) ≥ 1 and *p*adj < 0.05.

### Gene Ontology Enrichment Analysis

The gene ontology (GO) enrichment analysis of differentially expressed transcripts in mango pulp samples was performed using the KOBAS 2.0, GOseq, and GO database^1^.

### Correlation Analysis of Volatiles and Transcriptome Profile

The correlation analyses of differential volatiles and differential expression transcripts are achieved using the Pearson correlation analysis (SPSS version 15.0).

## Results

### Volatile Profiles in Mango at Different Development and Storage Stages

**FIGURE 1:**
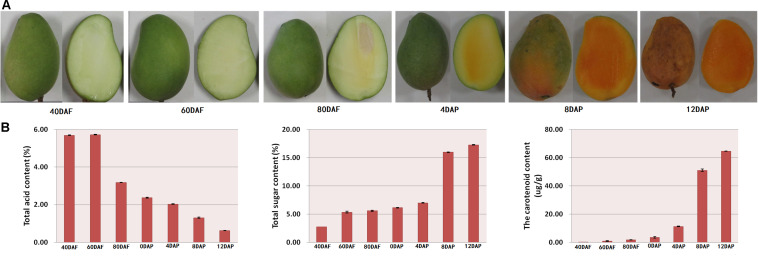
**(A)** Morphology of mango at different developmental stages and **(B)** total acid content, total sugar content, and total carotenoid content of mango peel. Samples labeled 40 DAF, 60 DAF, and 80 DAF are the peel samples of mango harvested at 40, 60, 80, and 90 days after the flowering, respectively; samples labeled 0 DAP, 4 DAP, 8 DAP, and 12 DAP are the peel samples of mango harvested at 90 days after flowering which kept for 0, 4, 8, and 12 days, respectively. DAF, days after flowering; DAP, days after picking.

The mango morphology was observed. Total acid, total sugar, and carotenoid contents of the fruit pulp were determined at different stages of fruit development and storage. As shown in Figure 1A, the mangoes were immature and green in color at 40, 60, and 80 days after flowering (DAF). Days after postharvest storage, the peel turned yellow, especially after 4 days of storage. Moreover, the pulps of mango harvested after 40 and 60 days of flowering looked light-green in color. The inner mesocarp of mango started to turn yellow on day 80 (Figure 1A). During the postharvest storage period, especially at 8 and 12 days after picking (DAP), the mango pulp turned orange (Figure 1A). The color hues were determined using a colorimeter (data not shown).

As shown in Figure 1B, there is a gradual decline in total acid content during the fruit development. The total acid content started to reduce after 60 DAF. The total acid content of the pulp samples of 80 DAF dropped to almost half compared to the value determined for pulp samples of 60 DAF. A linear decrease in total acid content was found for the mango pulp samples during the 12 days of storage. Also, there was a gradual decline in total acid content in the developing fruits after 60 DAF (Figure 1B). The total acid content was higher than 5.6% at 40 and 60 DAF. However, the total acid content was 0.63% at 12 DAP (Figure 1B). Moreover, the content of total sugar and total carotenoid gradually increased with the fruits ripening. There was a higher increase in total acid content of the pulp samples of 8 DAP (Figure 1B).

### Differential Analysis of Volatiles Among Different Development and Storage Stage

**FIGURE 2:**
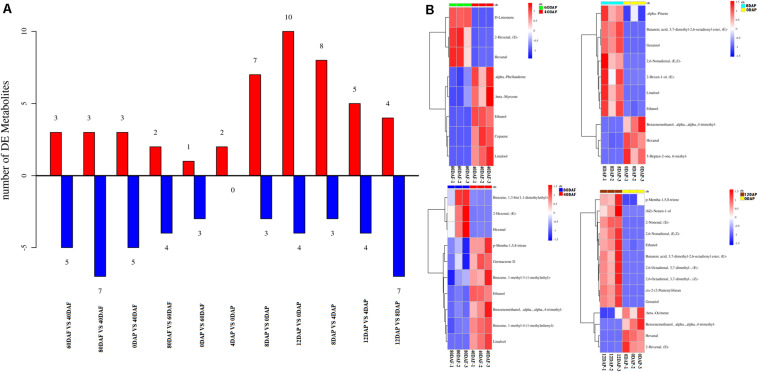
**(A)** The numbers of volatiles (metabolites) in mango pulp samples at different developmental and ripening stages and **(B)** Hierarchical clustering analysis of the differential volatiles in the samples. Samples labeled 40 DAF, 60 DAF, and 80 DAF are the peel samples of mango harvested at 40, 60, and 80 days after the flowering, respectively; samples labeled 0 DAP, 4 DAP, 8 DAP, and 12 DAP are the peel samples of mango harvested at 90 days after flowering which kept for 0, 4, 8, and 12 days, respectively. DAF, days after flowering; DAP, days after picking.

Volatile profiles of mango pulp samples were determined based on the headspace solid-phase microextraction method and GC-MS. The differential volatiles in mango pulp samples of different groups were screened according to the following criteria: fold change ≥ 1.5 or fold change ≤ 0.67; VIP≥ 1. The numbers of significantly different volatiles in each group are shown in Figure 2A. The result showed that significant differences were found for the numbers of differential volatiles between the two groups of samples (*p* < 0.05). However, the highest significant difference in the numbers of differential volatiles was determined between 12 DAP and 0 DAP samples (*p* < 0.01).

The data determined for the 12 DAP and 0 DAP group included ten up-regulated volatiles and four down-regulated volatiles. As shown in Figure 2B, three up-regulated volatiles determined for the 60 DAF vs. 40 DAF group were *D*-limonene, (*E*)-2-hexenal, and hexanal; three up-regulated volatiles found in the 80 DAF vs. 40 DAF group were (*E*)-2-hexenal, hexanal, and 1,3-bis(1,1-dimethylethyl)-benzene; ten up-regulated volatiles determined in the 12 DAP and 0 DAP group were (2*E*)-nonenal, (2*E*)-(2-pentenyl)furan, geraniol, (*E*,*Z*)-2,6-nonadienal, ethanol, butanoic acid, *p*-mentha-1,5,8-triene, (*E*)-3,7-dimethyl-2,6-octadienal, (*Z*)-3,7-dimethyl-2,6-octadienal, and (6*Z*)-nonen-1-ol; seven up-regulated volatiles determined in the 8 DAP vs. 0 DAP group were (*E*)-butanoic acid 3,7-dimethyl-2,6-octadienyl ester, geraniol, linalool, ethanol, (*E*,*Z*)-2,6-nonadienal, α-pinene, and (*E*)-2-hexen-1-ol. These up-regulated volatiles might be the dominating aromatic components of Tainong mango.

As shown in Dataset 1, 181 volatiles were isolated and identified. The major volatiles detected in the ripen fruit samples are α-pinene, β-phellandrene, β-ocimene, *D*-limonene, β-myrcene, γ-terpinene, 2-carene, 3-carene, 4-carene, copaene hexanal, and (*E*)-2-hexenal (Dataset 1). In this study, the amounts of (*E*)-2-nonenal, (2*E*)-(2-pentenyl)furan, geraniol, (*E*,*Z*)-2,6-nonadienal, ethanol, butanoic acid, *p*-mentha-1,5,8-triene, (*E*)-3,7-dimethyl-2,6-octadienal, (*Z*)-3,7-dimethyl-2,6-octadienal, and (6*Z*)-nonen-1-ol in the mango pulp samples of 12 DAP were higher than that of the sample at baseline (0 DAP) (Figure 2B), whereas the amounts of (*E*)-3,7-dimethyl-2,6-octadienyl-butanoate, geraniol, linalool, ethanol, (*E*,*Z*)-2,6-nonadienal, α-pinene, and (*E*)-2-hexen-1-ol in the mango pulp samples of 8 DAP were higher than the sample at baseline.

### RNA Sequencing Analysis of Mango During Different Development and Storage Stages

Molecular insight of volatile biosynthesis in mango pulp during the fruit development and storage can be obtained from RNA sequencing (RNA-Seq) analysis. Seven cDNA of the fruit samples, collected after 40, 60, 80, and 90 DAF, and at 4, 8, and 12 days of storage were constructed and large-scale sequenced. 47.29-79.61 million raw reads and 46.48-78.33 million cleaned reads were generated in each sample (Table 1). The Q20 (the percentage of bases with a Phred score greater than 20) and Q30 (the percentage of bases with a Phred score greater than 30) were higher than 95% (Table 1).

**TABLE 1:** Reads numbers, Q20 values, Q30 values, and gas chromatography (GC) content of mango pulp based on the RNA sequencing (RNA-Seq) data in the libraries of A, B, C, D, E, F, and G.

Sample	Raw reads	Clean reads	Q20 (%)	Q30 (%)	GC content (%)	Total mapped reads
A_1	54542030	53752304	98.71	95.68	45.04	41320416 (76.87%)
A_2	79607262	78327428	98.68	95.59	44.67	60458266 (77.19%)
A_3	66307584	65005808	98.69	95.6	44.67	50076660 (77.03%)
B_1	61825070	60940514	98.55	95.17	44.91	47642222 (78.18%)
B_2	61776038	60874120	98.71	95.66	44.81	47623800 (78.23%)
B_3	62783660	61896096	98.72	95.65	44.75	48219096 (77.90%)
C_1	65584430	64443166	98.74	95.73	44.65	50675552 (78.64%)
C_2	66911796	65761656	98.67	95.52	44.68	51653690 (78.55%)
C_3	70930988	69608456	98.58	95.29	44.2	54910004 (78.88%)
D_1	64323328	63325728	98.66	95.46	44.22	48845080 (77.13%)
D_2	53106480	52366662	98.73	95.72	44.51	40206360 (76.78%)
D_3	61134368	60176382	98.66	95.47	44.35	46130222 (76.66%)
E_1	64246102	63464652	98.69	95.53	44.45	49456492 (77.93%)
294E_2	47298170	46481888	98.68	95.48	44.36	36320930 (78.14%)
E_3	58093350	57090472	98.75	95.69	44.49	44561666 (78.05%)
F_1	59865570	58791448	98.55	95.12	44.19	45797898 (77.90%)
F_2	67186956	66240678	98.75	95.71	44.1	51789204 (78.18%)
F_3	60845708	59572020	98.72	95.6	44.13	46554538 (78.15%)
G_1	73520470	72003528	98.78	95.73	43.98	56418766 (78.36%)
G_2	54733094	53300870	98.79	95.77	44.01	42180466 (79.14%)
G_3	68004602	67124008	98.65	95.35	43.96	-

*A, 40 DAF; B, 60 DAF; C, 80 DAF; D, 0 DAP; E, 4 DAP; F, 8 DAP; G, 12 DAP. _1, _2, and _3 represent the triplicate analyses of each sample. Q20 is the percentage of bases with a Phred score greater than 20, whereas Q30 is the percentage of bases with a Phred score greater than 30.*

After assembled using the Trinity software, the transcripts of each sample were acquired. The frequency and numbers of transcripts and unigene in the corresponding length are shown in Supplementary Figure 1A. The CDs were predicted by comparing them with NR protein library, Swissprot protein library, and ESTScan v3.0.3 software. The counts of CDs with different lengths were also predicted based on the corresponding method (Supplementary Figure 1B).

**FIGURE 3:**
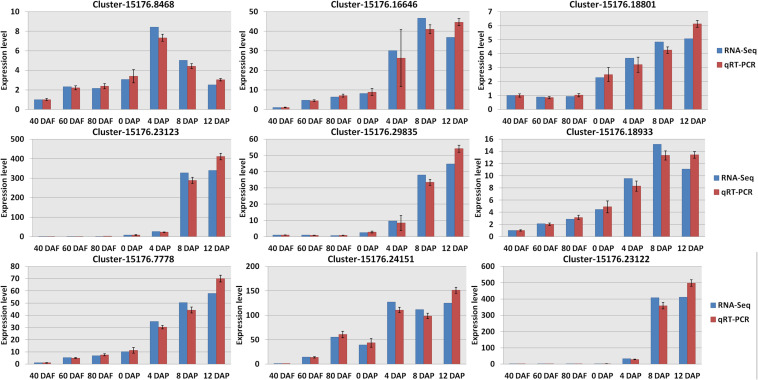
Verification the RNA sequencing (RNA-Seq) results by real-time quantitative PCR (qRT-PCR). All data were presented as mean ± standard error of the mean of triplicate analyses. DAF, days after flowering; DAP, days after picking. Gene names (Supplementary Table 2): Cluster-15176.8468 (wrbA); Cluster-15176.23123 (LOX1_5); Cluster-15176.7778 (accA); Cluster-15176.16646 (4CL); Cluster-15176.29835 (SXD1); Cluster-15176.24151 (fabI); Cluster-15176.18801 (VTE1); Cluster-15176.18933 (TAT); and Cluster-15176.23122 (LOX1_5).

Hierarchical clustering analysis of Pearson correlation according to the level of gene expression levels revealed that a high correlation was found for the gene expression in mango pulp samples between different development stages (Supplementary Figure 1C). Besides the three replicates of mango samples collected after 40 days of flowering, the Pearson correlation coefficients among the three repetitions of the other samples collected were higher than 0.95, which showed good reliability and repeatability of RNA-Seq data. Verification of the RNA-Seq data was done based on the nine selected genes (Figure 3 and Dataset 2).

### Differentially Expressed Transcripts and Gene Ontology Enrichment Analysis

**FIGURE 4:**
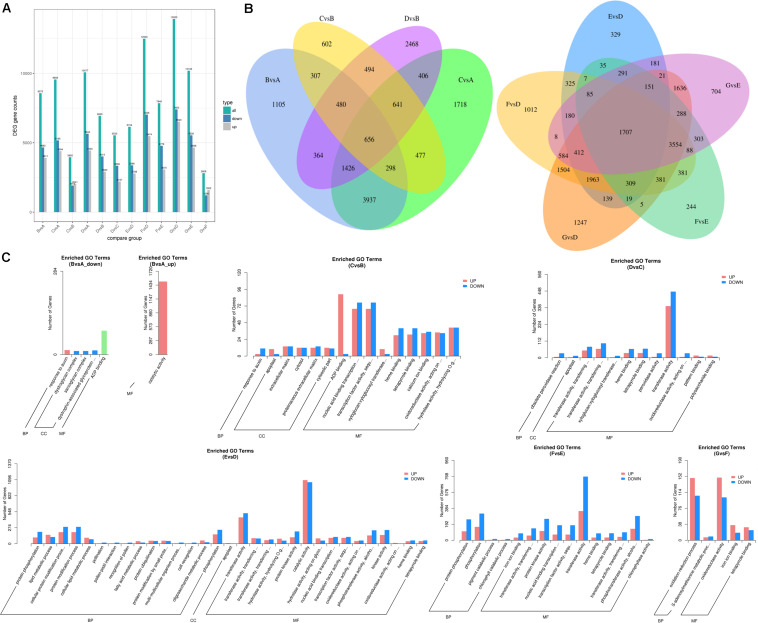
Differentially expressed transcripts of mango pulp samples at different developmental and ripening stages. **(A)** Total numbers of the differentially expressed genes (DEGs), up-regulated genes, and down-regulated genes between different samples, B vs. A, C vs. A, D vs. A, C vs. B, D vs. B, D vs. C, E vs. D, F vs. D, G vs. D, G vs. E, F vs. E, and G vs. F; **(B)** Venn diagram of the DEGs between different samples, B vs. A, C vs. A, C vs. B, D vs. B (left) and E vs. D, F vs. D, G vs. D, G vs. E, F vs. E (right); **(C)** GO classification of the up-regulated genes and down-regulated genes between different samples, B vs. A, C vs. B, D vs. C, E vs. D, F vs. E, and G vs. F; A–C are the samples collected after 40, 60, and 80 days of flowering, respectively; D–G are the samples stored for 0, 4, 8, and 12 days which collected after 90 days of flowering.

The differentially expressed transcripts in the groups of 60 DAF vs. 40 DAF, 80 DAF vs. 40 DAF, 90 DAF vs. 40 DAF, 80 DAF vs. 60 DAF, 90 DAF vs. 60 DAF, 90 DAF vs. 80 DAF, 4 DAP vs. 0 DAP, 8 DAP vs. 0 DAP, 12 DAP vs. 0 DAP, 8 DAP vs. 4 DAP, 12 DAP vs. 4 DAP, and 12 DAP vs. 8 DAP were identified based on the DEGSeq analysis for obtaining an overview of the interesting genes. The numbers of differentially expressed transcripts in each group are shown in Figure 4A and Dataset 2. The results showed that the most differentially expressed transcripts were found in the group of 12 DAP vs. 0 DAP (including 6,520 up-regulated transcripts and 7,400 down-regulated transcripts), where the data are in line with the results obtained from the determination of differential volatiles (Figure 2A). The Venn diagram also shows that 24 mutually differentially expressed transcripts existed in 60 DAF vs. 40 DAF, 80 DAF vs. 40 DAF, 80 DAF vs. 60 DAF, and 90 DAF vs. 60 DAF, whereas the number of commonly differentially expressed transcripts in 4 DAP vs. 0 DAP, 8 DAP vs. 0 DAP, 12 DAP vs. 0 DAP, 8 DAP vs. 4 DAP, and 12 DAP vs. 4 DAP (Figure 4B). These differentially expressed transcripts might be relevant to the mango development and formation of aromatic compounds.

The GO analysis was performed to show the comprehensiveness of functions of differentially expressed transcripts. As shown in Figure 4C, the gene functions are described based on the cellular components, molecular functions, and biological processes. During the fruit development, the most enriched GO terms for the groups of 60 DAF vs. 40 DAF and 90 DAF vs. 80 DAF were catalytic activity and transferase activity, respectively. Adenosine diphosphate (ADP) binding and transcription factor activity were the two most enriched GO terms for 80 DAF vs. 60 DAF. During storage of mango, the most enriched GO terms found were catalytic activity (4 DAP vs. 0 DAP), transferase activity (8 DAP vs. 4 DAP), and oxidoreductase activity (12 DAP vs. 8 DAP).

### Correlation Analysis of Differential Volatiles and Differentially Expressed Transcripts

**FIGURE 5:**
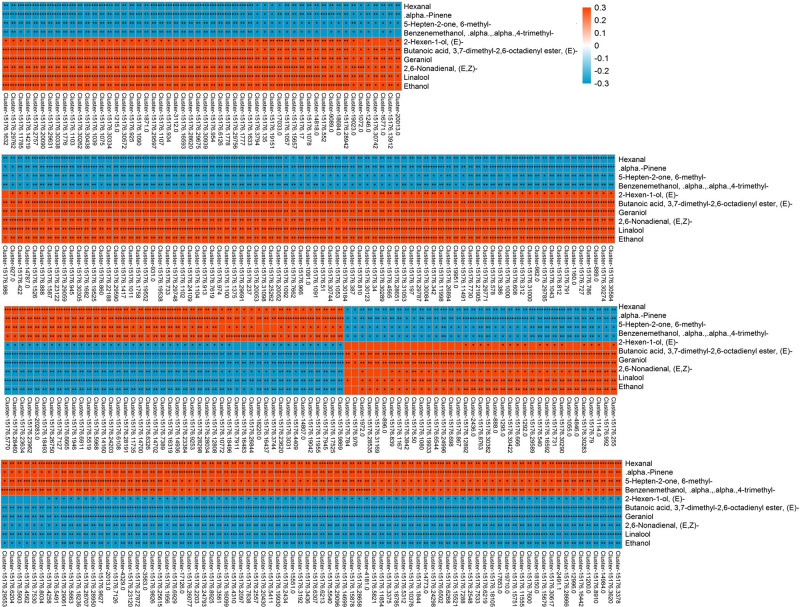
Correlation analysis of transcriptome and volatile profiles of mango pulp samples from the 8 DAP vs. 0 DAP group. The X-coordinate is the transcript name, and the Y-coordinate is the metabolite name. Each square represents the correlation and significance of the transcript and the metabolite. The correlation coefficient is presented based on different colors and shades. Red represents a positive correlation and blue represents a negative correlation. The darker the color, the higher the correlation. The names of the identified compounds and their related unigene-id are tabulated in Supplementary Table 1.

Correlation analysis was performed to explore the transcripts related to aroma biosynthesis during the development and storage of Tainong mango (Figure 5, Dataset 3). The results showed that 304 differentially expressed transcripts determined in the group of 8 DAP vs. 0 DAP were either positively or negatively correlated with 3,7-dimethyl-2,6-octadienyl ester, (*E*)-butanoic acid, geraniol, hexanal, 6-methyl-5-hepten-2-one, linalool, ethanol, α,α,4-trimethyl-benzenemethanol, (*E,Z*)-2,6-nonadienal, α-pinene, and (2*E*)-hexen-1-ol; 420 differentially expressed transcripts determined in the group of 4 DAP vs. 0 DAP were either positively or negatively correlated with α,α,4-trimethyl-benzenemethanol and (*E,Z*)-2,6-nonadienal; 601 differentially expressed transcripts determined in the group of 12 DAP vs. 0 DAP were either positively or negatively correlated with (2*E*)-nonenal, hexanal, (2*E*)-(2-pentenyl)furan, geraniol, (2*E*)-hexenal, (*E*,*Z*)-2,6-nonadienal, ethanol, (*E*)-3,7-dimethyl-2,6-octadienal, (*Z*)-3,7-dimethyl-2,6-octadienal, butanoic acid, α,α,4-trimethyl-benzenemethanol, (6*Z*)-nonen-1-ol, p-mentha-1,5,8-triene, and β-ocimene; 277 differentially expressed transcripts determined in the group of 80 DAF vs. 60 DAF were either positively or negatively correlated with α,α,4-trimethyl-benzenemethanol, 1-(4-methylphenyl)- ethanone, 1-methyl-4-(1-methylethenyl)-benzene, p-mentha-1,5,8-triene, alloaromadendrene, and 3-hexenal; 381 differentially expressed transcripts determined in the group of 60 DAF vs. 40 DAF were either positively or negatively correlated with D-limonene, ethanol, copaene, linalool, α-phellandrene, (2*E*)-hexenal, hexanal, and β-myrcene (Dataset 3).

### Genes and Enzymes Related to the Metabolism of Aromatic Compounds

**FIGURE 6:**
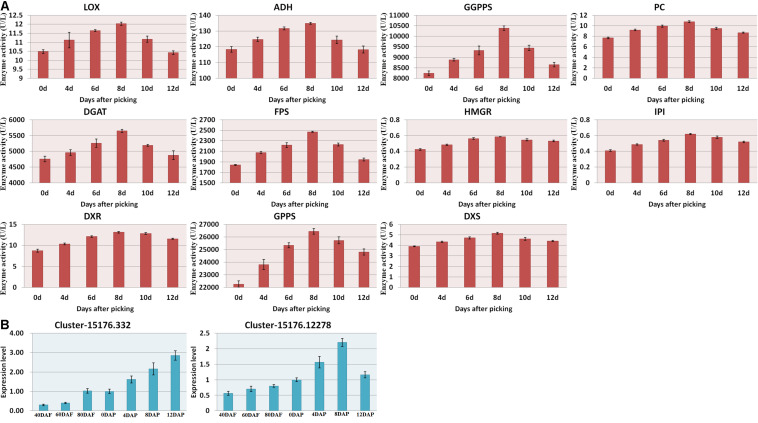
**(A)** Enzyme activities of the mango pulp samples and **(B)** gene expressions of E5.5.1.13 (Cluster-15176.332) and KAO (Cluster-15176.12278) in the samples during the fruit development and ripening. The enzyme activities of 1-deoxyd-D-xylose-5-phosphate reductase (DXS), 1-deoxyxylose-5-phosphate synthase (DXR), geranyl pyrophosphate synthetase (GPPS), geranyl geranyl pyrophosphate synthetase (GGPPS), pyruvate carboxylase (PC), diacylglyceryltransferase (DGAT), farnesyl diphosphate synthase (FPS), and hydroxymethyl glutarate monoacyl CoA reductase (FPS) in the mango pulp samples stored at different days were determined using ELISA kits. All data are presented as means ± standard errors of the means. Triplicate analyses were performed for each sample. DAF, day after flowering; DAP, days after picking.

As shown in Figure 2B, the amount of α-pinene in the fruit sample stored on day 8 was significantly higher than that on the other days (*p* < 0.05). It indicated that α-pinene is one of the key aroma components of mango. The results showed that a total of eight genes involved in the diterpenoid metabolism pathways (Figure 6 and Supplementary Table 3). The transcriptome analysis manifested that the genes, such as Cluster-15176.332 (E5.5.1.13) and Cluster-15176.12278 (KAO), were expressed at the highest levels in the mango pulp samples of 8 DAP. The high expressions of Cluster-15176.3381 (E1.14.11.13), Cluster-15176.3380 (E1.14.11.13), Cluster-19253.0 (GA3,CYP701), Cluster-15991.0 (E1.14.11.13), Cluster-3324.0 (E1.14.11.13), and Cluster-15176.1075 (CYP82G1) were also detected in mango pulp samples during the fruit development and ripening. These genes involved in diterpenoid biosynthesis. The qRT-PCR analysis also verified the highest expressions of E5.5.1.13 (ent-copalyl diphosphate synthase) and KAO (ent-kaurenoic acid hydroxylase) in the samples of 8 DAP compared with the other stages. These enzymes have been reported to regulate the biosynthesis of gibberellin (Su et al., 2016; Szymczyk et al., 2020). Only one gene was found to be involved in monoterpenoid biosynthesis. It was K15095 (Cluster-4351.0). The gene is related to the biosynthesis of nerolidol in the fruit. However, nerolidol was not detected in the fruit samples.

The gene names of the respective UniGene IDs are depicted in Table 2. Ent-kaurene oxidase, gibberellin 2-β-dioxygenase, and gibberellin 2-β-dioxygenase 8 isoform X3 were highly expressed during the fruit development and ripening. Only ent-kaurenoic acid oxidase 1-like was highly expressed in the matured fruit, and it was the main enzyme expressed in the terpenoid metabolism. Moreover, 18 others genes detected were known to be involved in ubiquinone and other terpenoid-quinone biosynthesis. Gibberellin is known to be synthesized via terpenoid biosynthesis pathway (Boba et al., 2020), and kaurene oxidase catalyzes the gibberellin biosynthesis. Besides, ent-kaurene is a tetracyclic hydrocarbon precursor for gibberellins. Moreover, the enzyme activities measured using ELISA kits, including DXS, DXR, GPPS, GGPPS, PC, DGAT, FPS, and HGMR were found to be increased gradually during the storage, and generally attained maximum levels at 8 DAP (Figure 6A).

**TABLE 2:** UniGene ID of the transcripts and compounds identified in the mango pulp samples.

UniGene ID	Gene details
Cluster-15176.1075	GAY40054.1| hypothetical protein
Cluster-15176.3381	KDP37976.1| hypothetical protein
Cluster-15176.3380	XP_015574955.1| gibberellin 2-beta-dioxygenase
Cluster-15991.0	PSS05904.1| gibberellin 2-beta-dioxygenase
Cluster-19253.0	XP_006431588.1| ent-kaurene oxidase
Cluster-15176.332	ESR57297.1| ent-copalyl diphosphate synthase
Cluster-3324.0	XP_006467978.1| gibberellin 2-beta-dioxygenase 8 isoform X3
Cluster-15176.12278	XP_021808327.1| ent-kaurenoic acid oxidase 1-like,
Cluster-15176.13875	GAV73636.1| patatin domain-containing protein
Cluster-15176.7685	PIA28198.1| hypothetical protein
Cluster-15176.7686	EEF47638.1| diacylglycerol cholinephosphotransferase
Cluster-8978.0	XP_006433476.1| phospholipase D zeta 2 isoform X2
Cluster-15176.21081	XP_022878380.1| choline..ethanolaminephosphotransferase 1-like
Cluster-15176.17421	OAY57973.1| hypothetical protein
Cluster-15176.14147	XP_024036163.1| phospholipase D alpha 1
Cluster-15176.27952	KDO81980.1| hypothetical protein
Cluster-15176.22073	ESR39921.1| hypothetical protein
Cluster-15176.23120	AQZ55551.1| 9-lipoxygenase
Cluster-15176.23122	AQZ55556.1| linoleate 9S-lipoxygenase
Cluster-15176.23123	ANF89411.1| linoleate 9S-lipoxygenase
Cluster-15176.19505	ESR33138.1| hypothetical protein
Cluster-15176.23124	AQZ55551.1| 9-lipoxygenase
Cluster-15176.13300	ESR59166.1| hypothetical protein
Cluster-15176.23657	GAV90434.1| adh_short domain-containing protein
Cluster-15176.4077	ESR36401.1| hypothetical protein
Cluster-15176.7778	GAV68539.1| acetyl-CoA carboxylase carboxyl transferase subunit alpha
Cluster-15176.5176	XP_021897331.1| acetyl-coenzyme A carboxylase carboxyl transferase subunit alpha
Cluster-15176.21236	AIU99499.1| hydroxyacyl-ACP dehydrase
Cluster-15176.15013	GAY44796.1| hypothetical protein
Cluster-15176.19615	KDO86915.1| hypothetical protein
Cluster-15176.19806	KRG93630.1| hypothetical protein
Cluster-15176.19807	OMP10121.1| biotin..lipoyl
Cluster-15176.13810	XP_006488591.1| malonyl CoA-acyl carrier protein transacylase
Cluster-15176.23708	GAY52806.1| hypothetical protein
Cluster-15176.8090	KVI00409.1| Fatty acid desaturase
Cluster-15176.15658	ESR56644.1| hypothetical protein
Cluster-15176.20399	OWM86900.1| hypothetical protein
Cluster-15176.6573	GAY39911.1| hypothetical protein
Cluster-15176.17613	XP_006492369.1| long chain acyl-CoA synthetase 1
Cluster-15176.1088	PON38544.1| fatty acid desaturase
Cluster-15176.8360	XP_006482734.1| biotin carboxyl carrier protein of acetyl-CoA carboxylase 2
Cluster-15176.27073	KJB64462.1| hypothetical protein
Cluster-15176.27077	XP_006434188.1| long chain acyl-CoA synthetase 9
Cluster-15176.27074	XP_008784201.1| long chain acyl-CoA synthetase 9
Cluster-15176.27075	GAY41784.1| hypothetical protein
Cluster-15176.11597	AOR17397.1| palmitoyl-acyl carrier protein thioesterase
Cluster-15176.16995	XP_024045530.1| 3-oxoacyl-[acyl-carrier-protein] synthase III
Cluster-15176.22688	ESR59166.1| hypothetical protein
Cluster-15176.13123	GAY44882.1| hypothetical protein
Cluster-15176.16081	XP_022757277.1| acetyl-coenzyme A carboxylase carboxyl transferase
Cluster-15176.21237	XP_006492623.1| 3-hydroxyacyl-[acyl-carrier-protein] dehydratase
Cluster-15176.15551	AKA09592.1| stearoyl-ACP desaturase
Cluster-15176.15554	GAY44796.1| hypothetical protein
Cluster-15176.17196	GAV92051.1| acyl-ACP_TE domain-containing protein
Cluster-15176.7526	XP_021284424.1| stearoyl-[acyl-carrier-protein] 9-desaturase 6
Cluster-15176.24109	ESR56805.1| hypothetical protein
Cluster-15176.16301	PON38544.1| fatty acid desaturase
Cluster-15176.24027	PPS18419.1| hypothetical protein
Cluster-15176.24028	XP_006480952.1| acetyl-coenzyme A carboxylase carboxyl transferase
Cluster-15176.18609	XP_009413949.1| long chain acyl-CoA synthetase 6
Cluster-15176.18608	PIN10227.1| long-chain acyl-CoA synthetases
Cluster-15176.15400	CBI26222.3| unnamed protein product
Cluster-15176.24150	XP_024443812.1| enoyl-[acyl-carrier-protein] reductase [NADH] 1
Cluster-15176.24151	XP_023914015.1| enoyl-[acyl-carrier-protein] reductase [NADH] 1
Cluster-15176.24152	AIS93131.1| enoyl-acyl carrier protein reductase [NAD+]
Cluster-15176.24153	POF25828.1| enoyl-[acyl-carrier-protein] reductase [NADH]
Cluster-4351.0	XP_006467945.1| (3S,6E)-nerolidol synthase 1-like
Cluster-15176.10743	PHU22294.1| acyl-[acyl-carrier-protein] desaturase
Cluster-15176.23658	GAY44036.1| hypothetical protein
Cluster-15176.14875	OMO53044.1| thiolase
Cluster-19268.0	XP_021679005.1| very-long-chain enoyl-CoA reductase-like
Cluster-15176.19912	ESR59974.1| hypothetical protein
Cluster-13603.0	XP_013702088.1| palmitoyl-monogalactosyldiacylglycerol delta-7 desaturase
Cluster-15176.9059	XP_021736703.1| palmitoyl-monogalactosyldiacylglycerol delta-7 desaturase
Cluster-15176.16917	AQZ55555.1| acyl-CoA-oxidase-1
Cluster-15176.6546	GAY56458.1| hypothetical protein
Cluster-15176.20306	AIE48274.1| omega-6 fatty acid desaturase
Cluster-15176.12892	AIC34706.1| omega-6 fatty acid desaturase
Cluster-15176.24984	KYP77364.1| chalcone synthase 1
Cluster-15176.2365	XP_006437739.2| leucoanthocyanidin reductase
Cluster-15176.2366	POE95046.1| leucoanthocyanidin reductase
Cluster-15176.17061	AIY25000.1| flavanone 3’-hydroxylase
Cluster-15176.4728	AIY24979.1| cinnamate 4-mono-oxygenase
Cluster-15176.15897	APR63682.1| p-coumarate 3-hydroxylase 3
Cluster-15176.9748	XP_021664632.1| 4-coumarate–CoA ligase
Cluster-15176.14112	OMO89673.1| oxoglutarate..iron-dependent dioxygenase
Cluster-15176.13889	AIY24995.1| flavanone 3-hydroxylase 2
Cluster-15176.6688	AIY25002.1| dihydroflavonol 4-reductase
Cluster-15176.2805	BAX25479.1| alkyldiketide-CoA synthase
Cluster-15176.19426	XP_022862158.1| 4-coumarate–CoA ligase-like 7
Cluster-15176.1022	KDO60238.1| hypothetical protein
Cluster-15176.4870	XP_021294689.1| 4-coumarate–CoA ligase 1-like
Cluster-19931.0	KDO46519.1| hypothetical protein
Cluster-15176.18933	TAT| tyrosine aminotransferase
Cluster-15176.29835	AAP97931.1| tocopherol cyclase
Cluster-15176.18801	XP_018842576.1| tocopherol cyclase
Cluster-15176.8468	PSR96325.1| NAD(P)H dehydrogenase FQR1-like
Cluster-15176.1733	PRQ41134.1| putative 1,4-dihydroxy-2-naphthoyl-CoA synthase
Cluster-15176.12108	XP_011082392.1| probable NAD(P)H dehydrogenase (quinone) FQR1-like 3 isoform X3
Cluster-15176.6725	ESR60477.1| hypothetical protein
Cluster-15176.6726	GAY65474.1| hypothetical protein
Cluster-15176.6727	XP_022732769.1| probable aminotransferase
Cluster-15176.26169	PSR95337.1| nicotianamine aminotransferase
Cluster-15176.16646	XP_006488032.1| 4-coumarate–CoA ligase-like 7
Cluster-15176.7245	XP_006469904.1| tyrosine aminotransferase
Cluster-18980.0	XP_006484288.1| protein PHYLLO

## Discussion

### Volatile Components of Mango at Different Stages

Volatile components in mango account for the tempting aroma of the fruit, which is also the most important quality of mango pulp and processed mango products. In common with nutritional quality, texture, and color, and the aroma is generally shaped by the coordination of biochemical and developmental pathways (Fujisawa et al., 2013). Many volatile components have been isolated and identified in mature mango and its juice of different cultivars, and some aroma-contributing compounds have also been confirmed in the previous studies (Andrade et al., 2000; Lalel et al., 2003; Pino and Mesa, 2006; Shivashankara et al., 2006; Munafo et al., 2014; Zhang et al., 2019).

In line with a previous study, volatile profiles of Tainong mango of different stages of harvesting and storage, including three stages during the fruit development and four stages during storage, were determined. Literature shows that the transcriptomes of Alphonso mango pulp and flower collected from the seven stages of fruit development and ripening were determined (Deshpande et al., 2017a). The volatile profiling and transcriptome were integrated for the identification of genes related to the metabolism of volatiles.

A previous study reported that 4-hydroxy-2,5-dimethyl-3(2H)-furanone was an important aromatic compound detected in mango cultivars Haden, White Alfonso, Praya Sowoy, Royal Special, and Malindi (Munafo et al., 2014). However, the GC-MS data showed that the mango pulp samples had a low amount of 4-hydroxy-2,5-dimethyl-3(2H)-furanone. Ethyl octanoate, 3-carene, limonene, α-terpinene, α-terpinolene, hexanal, and *p*-cymene were also the key volatiles detected in the different cultivars of Australian mango (San et al., 2017). Some of these compounds, such as ethyl octanoate, terpinolene, and cymene, were not detected in Tainong mango. A total of 12 components, including 2,4-dimethylstyrene, were identified as the major aroma active compounds in Keitt mango juice (Zhang et al., 2019).

The mango stored at these two stages had a more intense aroma than the other five stages. It could be due to a gradual increase in the total sugar content and a gradual decrease in total acid content during fruit maturity (Figure 1B). The results also showed that 17 volatile components were the key aroma active compounds in Tainong mango, especially ethanol and (*E*,*Z*)-2,6-nonadienal. Moreover, three up-regulated volatiles in both groups of 60 DAF vs. 40 DAF and 80 DAF vs. 40 DAF, and about 180 volatile components identified in the mango pulp samples might be the potential aromatic compounds.

### Genes and Enzymes Involved in Metabolism of Aromatic Components

The transcriptome studies have put forth important information concerning the development of mango of different cultivars, such as Zill (Wu et al., 2014), Langra (Azim et al., 2014), Kent (Dautt-Castro et al., 2015), Dashehari (Srivastava et al., 2016), and Alphonso (Deshpande et al., 2017a). Most of these studies reported the general metabolic pathways involved in the biosynthesis of metabolites in the mango. The genes encoding multiple enzymes related to gluconeogenesis from carbohydrate metabolism, glycolysis, fatty acid biosynthesis and beta-oxidation, salicylic acid biosynthesis, citrate cycle, ethylene biosynthesis, amino acids biosynthesis and degradation, β-carotene biosynthesis, α-tocopherol biosynthesis, flavonoid biosynthesis, and terpenoid backbone synthesis have also been reported in the literature.

In this study, RNA-Seq was performed to explore the molecular mechanism of aroma compounds biosynthesis in mango during the fruit development, ripening, and storage. A large number of differentially expressed transcripts involved in multiple pathways have been identified among these samples. Quantitative RT-PCR analysis also showed that the relative expression patterns of eleven genes were consistent with the RNA-Seq data. The results indicate that the transcriptome data are reliable. The number of differentially expressed transcripts in the samples consisted of over 50,000 up-regulated and down-regulated transcripts. These genes are related to the development of mango. Only three genes were highly expressed and these genes are closely related to the biogenesis of aromatic components during the fruit development. These two genes, E5.5.1.13 and KAO, have never been reported in the transcriptome analysis and metabolic profiling of mango. Another gene that was also found to be highly expressed in the mango pulp samples of 8 DAP. K15095 was the only gene found to be highly expressed during the storage of mango.

A large number of transcription factors (TFs) has been identified in the fruit samples as these factors are known to regulate the fruit development and formation of the aroma of the fruit (Bastías et al., 2011; Hong et al., 2012; Shen et al., 2016; Liu et al., 2017; Lü et al., 2018; Zhang et al., 2018). These TFs might have been participated in controlling the mango development and the biosynthesis of aromatic components of the fruit. The contents of terpenes in mango have been quantified and reported in the literature (Pandit et al., 2009a,b). The expression of genes involved in GPP, FPP, and GGPP synthesis have also been studied (Azim et al., 2014; Wu et al., 2014; Dautt-Castro et al., 2015; Srivastava et al., 2016; Deshpande et al., 2017a). As reported by Ma et al. (2006), DXS, DXR, GPPS, GGPPS, PC, DGAT, FPS, and HGMRs are the key enzymes for terpenoid-isoprenoid biosynthesis. The results obtained from ELISA assays revealed that the activities of these enzymes increased gradually after harvested and reached a maximum level on day 8. On the contrary, the PCR analysis did not show expression of the genes related to these enzymes in the mango pulp samples. In this study, the two highly expressed genes are known to be involved in the pathways of diterpenoid biosynthesis.

## Conclusion

Mango is favorable due to its pleasant sensory quality and high nutritional values. Although some of the aromatic components have been identified in different cultivars of mango, little is known about the volatile profile in the Tainong mango. 181 volatiles were isolated and identified in fruits collected at seven stages. These components, especially ethanol and (*E*,*Z*)-2,6-nonadienal, were the key aroma active compounds in Tainong mango. RNA-Seq and comparative analysis showed a large number of DEGs during development and after picking. These involved in catalytic activity, transferase activity, ADP binding, transcription factor activity, and oxidoreductase activity. The content of α-pinene, expression of genes involved in terpenoid metabolism, and enzyme activities in terpenoid metabolic pathways gradually increased after picking and generally attained their maximum levels on day-8. The integrative analyses also revealed potential molecular insights into fruit development and aroma formation. This study provides important cues for future work on mango quality improvement.

## Data Availability Statement

The datasets presented in this study can be found in online repositories. The names of the repository/repositories and accession number(s) can be found below: (https://www.ncbi.nlm.nih.gov/), and the data accession number is PRJNA697524.

## Author Contributions

CL, MX, PY, and YT performed the experiments. CL conceived and designed the research. LL and JS analyzed data. MX and XH prepared the manuscript. CL, JS, and HEK checked and revised the manuscript. All authors contributed to the article and approved the submitted version.

## Conflict of Interest

The authors declare that the research was conducted in the absence of any commercial or financial relationships that could be construed as a potential conflict of interest.

## Footnotes

1 http://www.geneontology.org/

## Supplementary Material

Supplementary Figure 1 Overview the transcriptome analysis of mango pulp samples at different developmental and ripening stages. **(A)** The frequency and numbers of transcripts with different length; **(B)** The count of CDs with different length predicted by comparing with NR protein library and Swissprot protein library (left) or by estscan (3.0.3) software (right); **(C)** The hierarchical clustering analysis of Pearson correlation according to the level of gene expression levels in fruits of A, B, C, D, E, F, and G. A, 40 DAF; B, 60 DAF; C, 80 DAF; D, 0 DAP; E, 4 DAP; F, 8 DAP; G, 12 DAP. _1, _2 and _3 represent the Triplicate analyses of each sample.

## References

[B1] Andrade E. H. A. Maia J. G. S. Maria das Graças B. Z. (2000). Aroma volatile constituents of Brazilian varieties of mango fruit. *J. Food Compos. Anal.* 13 27–33. 10.1006/jfca.1999.0841

[B2] Azim M. K. Khan I. A. Zhang Y. (2014). Characterization of mango (*Mangifera indica* L.) transcriptome and chloroplast genome. *Plant Mol. Biol.* 85 193–208. 10.1007/s11103-014-0179-8 24515595

[B3] Bastías A. López-Climent M. Valcárcel M. Rosello S. Gómez-Cadenas A. Casaretto J. A. (2011). Modulation of organic acids and sugar content in tomato fruits by an abscisic acid-regulated transcription factor. *Physiol. Plant.* 141 215–226. 10.1111/j.1399-3054.2010.01435.x 21128945

[B4] Boba A. Kostyn K. Kozak B. Wojtasik W. Preisner M. Prescha A. (2020). Fusarium oxysporum infection activates the plastidial branch of the terpenoid biosynthesis pathway in flax, leading to increased ABA synthesis. *Planta* 251 1–14. 10.1007/s00425-020-03339-9 31950395

[B5] Boonbumrung S. Tamura H. Mookdasanit J. Nakamoto H. Ishihara M. Yoshizawa T. (2001). Characteristic aroma components of the volatile oil of yellow keaw mango fruits determined by limited odor unit method. *Food Sci. Technol. Res.* 7 200–206. 10.3136/fstr.7.200

[B6] Chauhan O. P. Raju P. S. Bawa A. S. (2010). “Mango flavor,” in *Handbook of Fruit and Vegetable Flavors*, ed. Hui Y. H. (Hoboken, NJ: Wiley), 319–343. 10.1002/9780470622834.ch19

[B7] Dautt-Castro M. Ochoa-Leyva A. Contreras-Vergara C. A. Pacheco-Sanchez M. A. Casas-Flores S. Sanchez-Flores A. (2015). Mango (*Mangifera indica* L.) cv. Kent fruit mesocarp de novo transcriptome assembly identifies gene families important for ripening. *Front. Plant Sci.* 6:62. 10.3389/fpls.2015.00062 25741352 PMC4332321

[B8] Deshpande A. B. Anamika K. Jha V. Chidley H. G. Oak P. S. Kadoo N. Y. (2017a). Transcriptional transitions in Alphonso mango (*Mangifera indica* L.) during fruit development and ripening explain its distinct aroma and shelf life characteristics. *Sci. Rep.* 7:8711. 10.1038/s41598-017-08499-5 28821734 PMC5562913

[B9] Deshpande A. B. Chidley H. G. Oak P. S. Pujari K. H. Giri A. P. Gupta V. S. (2017b). Isolation and characterization of 9-lipoxygenase and epoxide hydrolase 2 genes: Insight into lactone biosynthesis in mango fruit (*Mangifera indica* L.). *Phytochemistry* 138 65–75. 10.1016/j.phytochem.2017.03.002 28291596

[B10] Fujisawa M. Nakano T. Shima Y. Ito Y. (2013). A large-scale identification of direct targets of the tomato MADS box transcription factor RIPENING INHIBITOR reveals the regulation of fruit ripening. *Plant Cell* 25 371–386. 10.1105/tpc.112.108118 23386264 PMC3608766

[B11] Hong G. J. Xue X. Y. Mao Y. B. Wang L. J. Chen X. Y. (2012). Arabidopsis MYC2 interacts with DELLA proteins in regulating sesquiterpene synthase gene expression. *Plant Cell* 24 2635–2648. 10.1105/tpc.112.098749 22669881 PMC3406894

[B12] Kulkarni R. S. Chidley H. G. Pujari K. H. Giri A. P. Gupta V. S. (2012). Geographic variation in the flavour volatiles of Alphonso mango. *Food Chem.* 130 58–66. 10.1016/j.foodchem.2011.06.053

[B13] Lalel H. J. D. Singh Z. Tan S. C. (2003). Aroma volatiles production during fruit ripening of ‘Kensington Pride’ mango. *Postharvest Biol. Technol.* 27 323–336. 10.1016/S0925-5214(02)00117-5

[B14] Lebrun M. Plotto A. Goodner K. Ducamp M. N. Baldwin E. (2008). Discrimination of mango fruit maturity by volatiles using the electronic nose and gas chromatography. *Postharvest Biol. Technol.* 48 122–131. 10.1016/j.postharvbio.2007.09.010

[B15] Li B. Dewey C. (2011). RSEM: accurate transcript quantification from RNA-Seq data with or without a reference genome. *BMC Bioinformatics* 12:323. 10.1186/1471-2105-12-323 21816040 PMC3163565

[B16] Li X. B. Fan X. P. Wang X. L. Cai L. Yang W. C. (2005). The cotton ACTIN1 gene is functionally expressed in fibers and participates in fiber elongation. *Plant Cell* 17 859–875. 10.1105/tpc.104.029629 15722467 PMC1069704

[B17] Liao L. Dong T. Qiu X. Rong Y. Wang Z. Zhu J. (2019). Nitrogen nutrition is a key modulator of the sugar and organic acid content in citrus fruit. *PLoS One* 14:e0223356. 10.1371/journal.pone.0223356 31600253 PMC6786551

[B18] Liu H. Y. Cao X. M. Liu X. H. Xin R. Wang J. J. Gao J. (2017). UV-B irradiation differentially regulates terpene synthases and terpene content of peach. *Plant Cell Environ.* 40 2261–2275. 10.1111/pce.13029 28722114

[B19] Lü P. Yu S. Zhu N. Chen Y. R. Zhou B. Pan Y. (2018). Genome encode analyses reveal the basis of convergent evolution of fleshy fruit ripening. *Nat. Plants* 4 784–791. 10.1038/s41477-018-0249-z 30250279

[B20] Luo C. He X. H. Chen H. Hu Y. Ou S. J. (2013). Molecular cloning and expression analysis of four actin genes (MiACT) from mango. *Biol. Plant.* 57 238–244. 10.1007/s10535-012-0278-9

[B21] Ma L. Ding P. Yang G. He G. (2006). Advances on the plant terpenoid isoprenoid biosynthetic pathway and its key enzymes. *Biotechnol. Bull. 2006, S* 1 22–30.

[B22] Munafo J. P. Jr. Didzbalis J. Schnell R. J. Schieberle P. Steinhaus M. (2014). Characterization of the major aroma-active compounds in mango (*Mangifera indica* L.) cultivars Haden, White Alfonso, Praya Sowoy, Royal Special, and Malindi by application of a comparative aroma extract dilution analysis. *J. Agric. Food Chem.* 62 4544–4551. 10.1021/jf5008743 24766361

[B23] Pandit S. S. Chidley H. G. Kulkarni R. S. Pujari K. H. Giri A. P. Gupta V. S. (2009a). Cultivar relationships in mango based on fruit volatile profiles. *Food Chem.* 114 363–372. 10.1016/j.foodchem.2008.09.107

[B24] Pandit S. S. Kulkarni R. S. Chidley H. G. Giri A. P. Pujari K. H. Koöllner T. G. (2009b). Changes in volatile composition during fruit development and ripening of ‘Alphonso’ mango. *J. Sci. Food Agr.* 89 2071–2081. 10.1002/jsfa.3692

[B25] Pino J. A. (2012). Odour-active compounds in mango (*Mangifera indica* L. cv. Corazoìn). *Int. J. Food Sci. Technol.* 47 1944–1950. 10.1111/j.1365-2621.2012.03054.x

[B26] Pino J. A. Mesa J. (2006). Contribution of volatile compounds to mango (*Mangifera indica* L.) aroma. *Flavour Fragr. J.* 21 207–213. 10.1002/ffj.1703

[B27] San A. T. Joyce D. C. Hofman P. J. Macnish A. J. Webb R. I. Matovic N. J. (2017). Stable isotope dilution assay (SIDA) and HS-SPME-GCMS quantification of key aroma volatiles for fruit and sap of Australian mango cultivars. *Food Chem.* 221 613–619. 10.1016/j.foodchem.2016.11.130 27979249

[B28] Shen S. L. Yin X. R. Zhang B. Xie X. L. Jiang Q. Grierson D. (2016). CitAP2.10 activation of the terpene synthase CsTPS1 is associated with the synthesis of (+)-valencene in ‘Newhall’ orange. *J. Exp. Bot.* 67 4105–4115. 10.1093/jxb/erw189 27194737 PMC5301923

[B29] Shi J. Zou X. Huang X. Zhao J. Li Y. Hao L. (2013). Rapid detecting total acid content and classifying different types of vinegar based on near infrared spectroscopy and least-squares support vector machine. *Food Chem.* 138 192–199. 10.1016/j.foodchem.2012.10.060 23265476

[B30] Shivashankara K. S. Isobe S. Horita H. Takenaka M. Shiina T. (2006). Volatile aromatic constituents of tree ripened and mature green ‘Irwin’ mango fruits during low temperature storage. *J. Jpn. Soc. Hortic. Sci.* 75 209–212. 10.2503/jjshs.75.209

[B31] Srivastava S. Singh R. K. Pathak G. Goel R. Asif M. H. Sane A. P. (2016). Comparative transcriptome analysis of unripe and mid-ripe fruit of *Mangifera indica* (var.“Dashehari”) unravels ripening associated genes. *Sci. Rep.* 6:32557. 10.1038/srep32557 27586495 PMC5009307

[B32] Su P. Tong Y. Cheng Q. Hu Y. Zhang M. Yang J. (2016). Functional characterization of ent-copalyl diphosphate synthase, kaurene synthase and kaurene oxidase in the *Salvia miltiorrhiza* gibberellin biosynthetic pathway. *Sci. Rep.* 6:23057. 10.1038/srep23057 PMC4789781 26971881

[B33] Szymczyk P. Szymańska G. Lipert A. Weremczuk-Je/.zyna I. Kochan E. (2020). Computer-aided saturation mutagenesis of *Arabidopsis thaliana* ent-copalyl diphosphate synthase. *Interdiscip. Sci.* 12 32–43. 10.1007/s12539-019-00342-x 31309397 PMC7007437

[B34] Tamura H. Boonbumrung S. Yoshizawa T. Varanyanond W. (2001). The volatile constituents in the peel and pulp of a green Thai mango, Khieo Sawoei cultivar (*Mangifera indica* L.). *Food Sci. Technol. Res.* 7 72–77. 10.3136/fstr.7.72

[B35] Wu H. X. Jia H. M. Ma X. W. Wang S. B. Yao Q. S. Xu W. T. (2014). Transcriptome and proteomic analysis of mango (*Mangifera indica* L.) fruits. *J. Proteomics* 105 19–30. 10.1016/j.jprot.2014.03.030 24704857

[B36] Zhang L. Zhang Q. Li W. Zhang S. Xi W. (2019). Identification of key genes and regulators associated with carotenoid metabolism in apricot (*Prunus armeniaca*) fruit using weighted gene coexpression network analysis. *BMC Genom.* 20:876. 10.1186/s12864-019-6261-5 31747897 PMC6865023

[B37] Zhang W. Dong P. Lao F. Liu J. Liao X. Wu J. (2019a). Characterization of the major aroma-active compounds in Keitt mango juice: comparison among fresh, pasteurization and high hydrostatic pressure processing juices. *Food Chem.* 289 215–222. 10.1016/j.foodchem.2019.03.064 30955605

[B38] Zhang Y. Yin X. Xiao Y. Zhang Z. Li S. Liu X. (2018). An ETHYLENE RESPONSE FACTOR-MYB transcription complex regulates furaneol biosynthesis by activating QUINONE OXIDOREDUCTASE expression in strawberry. *Plant Physiol.* 178 189–201. 10.1104/pp.18.00598 29987002 PMC6130037

