# Supplementary figures and images for: Dynamic Analyses of Transcriptome and Metabolic Profiling: Revealing Molecular Insight of Aroma Synthesis of Mango (Mangifera indica L. Var. Tainong)
Source: Front Plant Sci. 2021 May 7;12:666805. doi: 10.3389/fpls.2021.666805 (PMC8138435; doi:10.3389/fpls.2021.666805)

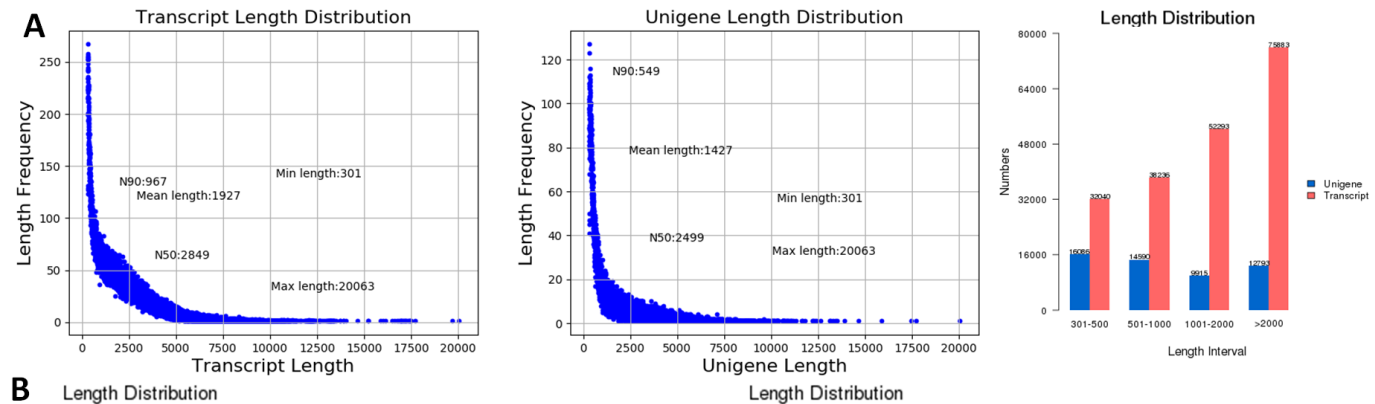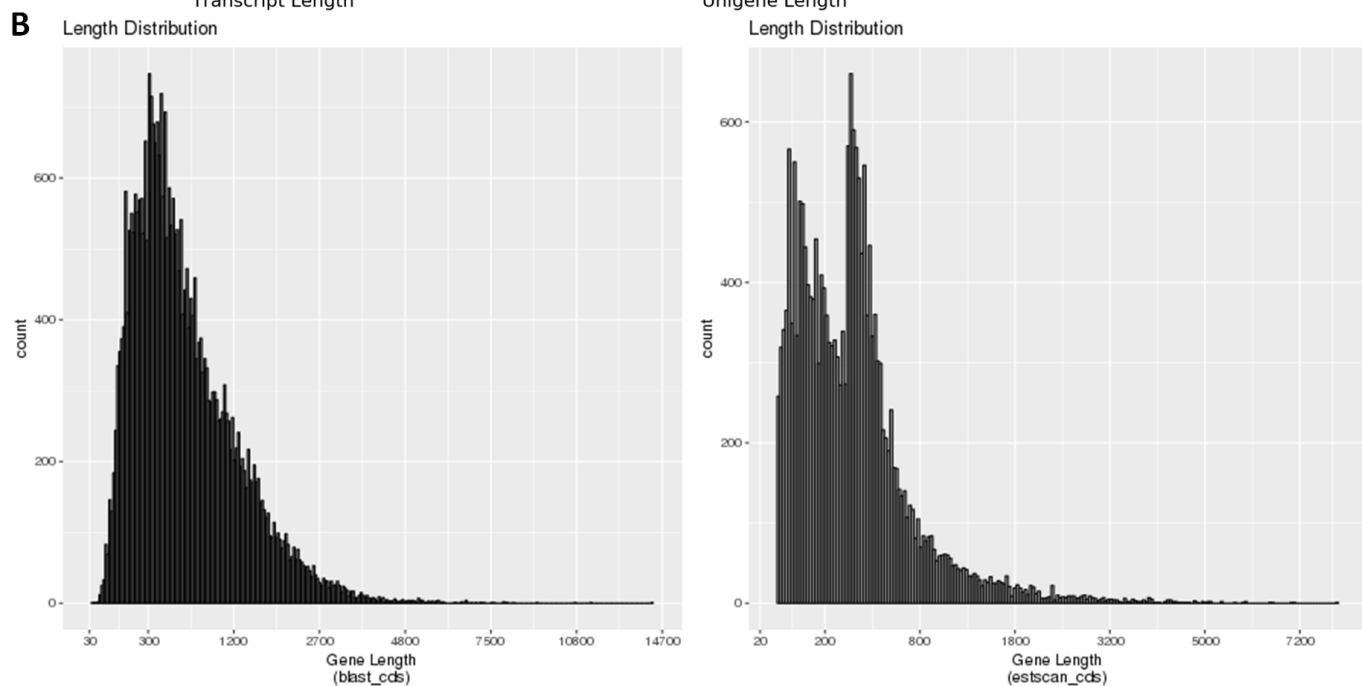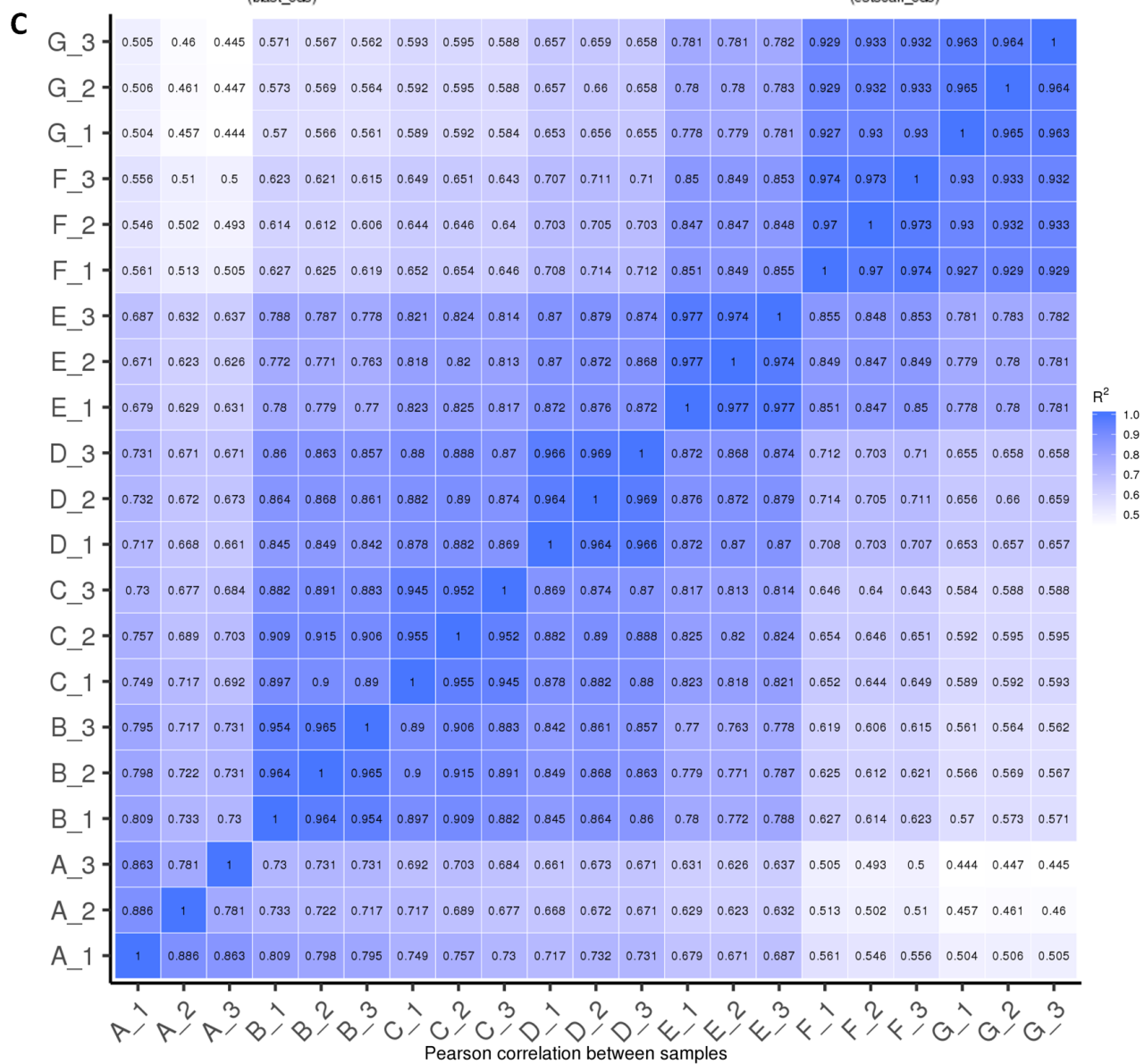

Supplement: Supplementary Figure 1 — Overview the transcriptome analysis of mango pulp samples at different developmental and ripening stages. (A) The frequency and numbers of transcripts with different length; (B) The count of CDs with different length predicted by comparing with NR protein library and Swissprot protein library (left) or by estscan (3.0.3) software (right); (C) The hierarchical clustering analysis of Pearson correlation according to the level of gene expression levels in fruits of A, B, C, D, E, F, and G. A, 40 DAF; B, 60 DAF; C, 80 DAF; D, 0 DAP; E, 4 DAP; F, 8 DAP; G, 12 DAP. _1, _2 and _3 represent the Triplicate analyses of each sample. [file Image_1.pdf]
