# Supplementary material for: Dynamic Analyses of Transcriptome and Metabolic Profiling: Revealing Molecular Insight of Aroma Synthesis of Mango (Mangifera indica L. Var. Tainong)
Source: Front Plant Sci. 2021 May 7;12:666805. doi: 10.3389/fpls.2021.666805 (PMC8138435; doi:10.3389/fpls.2021.666805)
Supplement: Supplementary file 2 [file Data_Sheet_1.pdf]

Table S1. List of primers used by RT-PCR analysis.

Gene ID	Forward primer	Length (bp)	Reverse primer	Length (bp)
Cluster-15176.332	TGCTTCCCACTTCATCAGC	19	ACATTAACCCACAGAACCACA	21
Cluster-15176.7686	ATCTTCCCTCTATGGATGCC	20	TGTTCGTCGTGCTTGCTT	18
Cluster-15176.14147	TGACTATTATCCTCCTATCCTTACC	25	CAGACACTTCCACGTCTTTGT	21
Cluster-15176.23122	GTCCCAGGTACATCTCATCCG	21	CCTCCCTAACCGTCCAACTA	20
Cluster-15176.23123	ACCAGTGGCTGATGAGTTGA	20	GTAATGTCTATACACCGTCAGAGC	24
Cluster-15176.23124	TCAACAAGGGTAATGCCAAGA	21	CCGCCCAAGTATCAGTCG	18
Cluster-15176.22073	TGCTTACAGGAGGAAACACC	20	GGACAAATCTGCCTGGCTAC	20
Cluster-15176.23657	CACAGTGGCAGGAGGTTAT	19	AGCCTAATGGAGGAGCAC	18
Cluster-15176.7778	CTCCATTTCGTTTCTCCTG	19	AACCGACAAGCAACTTCACTA	21
Cluster-15176.24151	CACCTCGCAAAGGGAAGC	18	GAACAGAAGGACCTAAACCACT	22
Cluster-15176.24152	CTTCCCACGCCGTAGACT	18	AGGATTGCCTGTTGATTTGA	20
Cluster-15176.16646	CCACGGTGTAAACAGGATT	19	CTTTCGCCATTTGGGTAT	18
Cluster-15176.18801	TCGAGGACTATGCGTTGC	18	TCATTCTCCGTATCACTCTTGT	22
Cluster-15176.18933	TTCAGCAGCCGCAAA	16	AAAGACGGAAACCAAACC	18
Cluster-15176.18933	TTCAGCAGCCGCAAA	16	AAAGACGGAAACCAAACC	18
Cluster-15176.8468	TCAGGCATCTCGGTTGG	17	GACGAATGGGAGACAGTAGAA	21
Cluster-15176.7245	GCAAACCCTAAACACGGAGA	20	ATGCCAAGAAGCAGTAAGAGC	21
Cluster-15176.29835	TGACAGCATTGGGATAAAGAA	21	AGCAGGTGGGTTGAGGC	17
Cluster-15176.12278	GTACGGAGTAGAGCACAATAGG	22	GTTTGGTTAAAGGCGTTAGAG	21
Cluster-15176.12278	GTACGGAGTAGAGCACAATAGG	21	GTTTGGTTAAAGGCGTTAGAG	22
MiACT	AATGGAACTGGAATGGTCAAGGC	23	TGCCAGATCTTCTCCATGTCATCCCA	26

Table S2. Gene information for the verification study.

Gene-ID	Gene Length	Gene Name	Description	KO ID
Cluster-15176.18801	3667	VTE1	tocopherol cyclase	K09834
Cluster-15176.18933	4298	TAT	tyrosine aminotransferase	K00815
Cluster-15176.23122	3165	LOX1_5	linoleate 9S-lipoxygenase	K15718
Cluster-15176.16646	5071	4CL	4-coumarate--CoA ligase	K01904
Cluster-15176.29835	2887	SXD1	tocopherol cyclase	K09834
Cluster-15176.24151	1825	fabI	enoyl-	K00208
Cluster-15176.8468	1555	wrbA	NAD(P)H dehydrogenase (quinone)	K03809
Cluster-15176.23123	789	LOX1_5	linoleate 9S-lipoxygenase	K15718
Cluster-15176.7778	1716	accA	acetyl-CoA carboxylase carboxyl transferase subunit alpha	K01962

Table S3. Gene information for the eight highly expressed genes.

Gene-ID	Gene Length	Gene Name	Description	NR Score
Cluster-15176.332	2737	E5.5.1.13	ent-copalyl diphosphate synthase	1452
Cluster-15176.12278	2763	KAO	ent-kaurenoic acid hydroxylase	1032
Cluster-15176.3380	1576	E1.14.11.13	gibberellin 2-oxidase	1012
Cluster-19253.0	912	GA3, CYP701	ent-kaurene oxidase	683
Cluster-15991.0	1686	E1.14.11.13	gibberellin 2-oxidase	888
Cluster-3324.0	1568	E1.14.11.13	gibberellin 2-oxidase	1283
Cluster-15176.1075	2113	CYP82G1	cytochrome P450, family 82, subfamily G, polypeptide 1	1966
Cluster-15176.3381	1371	E1.14.11.13	gibberellin 2-oxidase	1335
